# Supplementary material for: Livestock and rodents within an endemic focus of Visceral Leishmaniasis are not reservoir hosts for Leishmania donovani
Source: PLoS Negl Trop Dis. 2022 Oct 20;16(10):e0010347. doi: 10.1371/journal.pntd.0010347 (PMC9624431; doi:10.1371/journal.pntd.0010347)
Supplement: Attachment — Submitted filename: Comments-Reviewer.docx [file pntd.0010347.s001.docx]

The manuscript entitled **“**Domestic Mammals as potential Reservoir Hosts for *Leishmania donovani* in India” describes an interesting study. The results of this study are of major eco-epidemiological importance and should lead to a better control of visceral leishmaniasis in the Indian Subcontinent. The manuscript should be published after taking into consideration the following comments:

**Title:** This study concerned not only domestic animals but also rodents living in the peri-domestic areas. In addition, the results provided strong evidence that investigated mammals are not reservoir hosts for *Leishmania donovani*. Thus, the title is misleading and therefore it should be change to “Mammals of the peridomestic areas in an endemic focus of VL are not reservoir host for *Leishmania donovani”*

**Introduction**

Lines 72-73: The authors should differentiate between zoonotic visceral leishmaniasis due to *L. infantum* and anthroponotic visceral leishmaniasis due to *Leishmania donovani*.

Lines 102: In addition to canids, lagomorphs and wild rodents can also act as significant reservoirs.

**Methods**

I have a serious concern with the method.

Why the authors exclude dogs from the study? This is an important missing data.

48 hrs after blood feeding, engorged sandflies were stored in 70% alcohol for further DNA extraction and qPCR for *L. donovani*. The normal cycle of *L. donovani* within the sandfly takes 8-10 days to reach stationary phase. Why the authors did not allow for a full cycle accomplishment of *L. donovani* within the sandfly.

**Results**

Since xenodiagnosis was repeated throughout the year, the authors should report results at each time.

**Discussion**

The authors should report studies concerning xenodiagnoses performed on humans.

Taking into account that dogs are considered as reservoir hosts for *L. donovani* (Jumbulingam et al., 2017), the authors should discuss the rationale of not testing dogs.
