# Supplementary material for: Livestock and rodents within an endemic focus of Visceral Leishmaniasis are not reservoir hosts for Leishmania donovani
Source: PLoS Negl Trop Dis. 2022 Oct 20;16(10):e0010347. doi: 10.1371/journal.pntd.0010347 (PMC9624431; doi:10.1371/journal.pntd.0010347)
Supplement: Attachment — Submitted filename: PLOS NTD Reviewer Coments and Responses.docx [file pntd.0010347.s002.docx]

**Response to reviewers**

We thank the reviewers for their thoughtful and helpful comments which have improved the content of this manuscript. Our responses will occur in Garamond to allow them to stand out from reviewer comments.

Reviewer #1: Objectives are clearly stated.
The Study design does not allow testing infectiousness, and this is acknowledged by the authors in the discussion.
The study population is clearly described, but not the rationale for the sample size. Therefore, it is not possible to assess whether the selected sample size allows enough power to address the hypothesis.

Since xenodiagnosis has not previously been performed on non-human animals in VL endemic regions in India. regarding the true proportion of infective animal reservoirs is very low (<1/1000) in ISC (Singh et al., 2013). We interpret <2% infectivity as arguing against an important role for infected animals in maintaining the *L. donovani* transmission cycle. Using an exact one-sided binomial test at the 2.5% level, then with a sample size of n= 200, we have at least 90% power to show that the proportion infective is less than 2%.

In table 1, The sample size needed to give 90% power to show that the true proportion is greater than 0 given the true proportion is p. This is the same as the sample size needed to give 90% power to observe at least 1 infection out of n. As per the reviewer’s suggestion we have added the rationale for selecting the number of animals.

No concerns regarding ethical issues.

Reviewer #2: The authors should indicate in the methods how they decided on the number of sand flies for xenodiagnosis, and show that the number they used per animal species or group is sufficient to detect Leishmania infection, based on other xenodiagnosis studies.
See other and specific comments on the general comments.

See above explanation regarding sample size for Reviewer 1.

Reviewer #1: The results reflect the planned analysis and are clearly presented.

Reviewer #2: Results presented on tables are not mentioned in the text, and some results are only mentioned in the Discussion.

We have made changes in results and discussion part as suggested by reviewer in the revised manuscript.

See other and specific comments on the general comments.

Reviewer #1: The conclusions reflect the data obtained. And some limitations are stated. It is missed a proposed plan for similar analyses/studies in the future.

Reviewer #2: Discussion of the limitations should be improved. The conclusions rest on the capacity of the xenodiagnosis to detect transmission with the sample size used, which needs to be demonstrated.

We are thankful to the reviewers for the observation and we have made the necessary changes suggested by the reviewers in the revised manuscript.

See other and specific comments on the general comments.

**Summary and General Comments**
**Reviewer #1**:

A great progress has been made on the Indian sub-continent (ISC) to reach the targets set for visceral leishmaniasis (VL) in the WHO Road Map for NTDs, 2012-2020, i.e. Bangladesh, India and Nepal achieving elimination as a public health problem (<1 case/10 000 population, district or subdistrict level in endemic area). Pending validation, this target has been achieved by Bangladesh and Nepal, while India is very close (92%). In the new WHO Road Map for NTDs, 2021-2030 it is expected that the target will be reached by 2023.
With this situation, activities like the one proposed by the authors are key in order to assess that no residual foci of transmission remain that can revert the current situation in case control efforts are relaxed.
The study presented is pertinent and activities like this should be implemented across the ISC in order to gain understanding on the possibility of a zoonotic/anthropozoonotic cycle for *Leishmania donovani*.
There are however some points that in my opinion should be addressed/detailed in the manuscript in order to make this activity an example going forward and for others:

1) Xenodiagnosis, as it has been done, provides very little information. To assess infectivity to sand flies and the possibility of transmission of the parasites to another host, the screening for *Leishmania* parasites should have been conducted after blood meal digestion and defecation, which is beyond 48h. And ideally, infection should be confirmed by microscopy and observation of *Leishmania* parasites after dissecting the sand fly.

We are grateful to the reviewer for this observation. Studies on parasite development in sand fly gut shows that after 48hrs of infected blood meal the peritrophic membrane had broken down and many procyclic promastigotes and nectomonads were readily visible while by 72hrs, most of the bloodmeal had been digested and the bulk of the parasites had passed out with it. Although microscopy is gold standard method to see viable parasites it has limitations of low sensitivity. In recent artificial infection experiment in sand fly, no parasites were seen via microscopy at concentrations of <50,000 parasites/ml while qPCR detected parasites with infection concentration of 10,000 parasites/ml at 48–72hrs post infectious feed (Tiwary et al., 2017). This means if lower parasite burden infections were established in peri-domestic mammals may lead low level transmission to sand flies, would not be detected. Multiple xenodiagnosis studies have reliably used qPCR to identify transmission to sand flies We have revised our manuscript with these references.

2) Why the animals (livestock) were selected based on 'owner report of illness'? Why not including asymptomatic animals?

We are thankful to the reviewer for this valuable comment. In endemic villages animals are living in close proximity with humans and they may be exposed by bites of infected sand fly, but without diagnostic confirmation we could not identify them as subclinical animals. We sampled to identify *Leishmania* infection/ exposure and conducted xenodiagnosis simultaneously therefore we do not mention “asymptomatic animals” in selection criteria. We have now revised the sentence accordingly in the manuscript.

3) Selection of animals, what is the total (estimated) number of each of the species selected in the different subdistricts or sampling units? This figure provides the ecological context. Dogs were not included, despite being reservoirs for different Leishmania species, why?

The exact composition of animals within the cohort was based on the natural abundance and likelihood of reservoir status. Estimated sampling equal to 40 cow, 40 goats, 40 water buffalo and 60 members of the order rodentia that we randomly sampled across these areas (refer Figure 1)

We sincerely appreciate the reviewer's points on excluding dogs from study. Our initial objective was to assess the role of livestock and rodents in endemic areas in relation to transmission as livestock density can play an important role in *Leishmania* epidemiology. Livestock are an important part of households in endemic setting of ISC. We agree that dogs are key reservoir hosts for the zoonotic transmission cycle and other findings in ISC also concluded that dogs are infected with *Leishmania donovani* and could be reservoir host. We are actively working in this area including serology and molecular identification of *Leishmania* infection in dogs as well as clinical investigations and its relation to infectiousness. We wish to share our findings with the *Leishmania* research community very soon.

4) Preparation of sand flies: what is the rationale for selecting 30-35 female *Phlebotomus argentipes* and no more or less? Authors could refer to other xenodiagnosis studies using 'hosts' for which infection has been confirmed. Even in these cases the infectivity is low. Therefore, are 35 sand flies enough to test infectiousness of the selected animals?

We sincerely appreciate the reviewers for this observation. We agree with referring to other xenodiagnosis studies where infection was confirmed. A recent xenodiagnosis study conducted on asymptomatic human subjects to test their infectiousness where infectivity is very low also used 30 sand flies per feeding cup, as well as previous xenodiagnostis published by our group (Scorza et al., 2021, Schaut et al., 2015). Feeding success ranged from 5% to 65% depending on the blood source and number of flies in the feeding cup. The more flies per cup, the higher the blood-feeding success, maximized at ~30 females. Based on these results and previous studies it was decided to limit the number of females per feed to 30 and exposed 2 feeding cups (60-70 flies) per subject. We have made changes in revised manuscript with references.

5) Statistical analysis. There is no mention to the rationale for selecting the number of animals and sandflies used in the study.

The rationale for selecting specific numbers of different species of animals was already provided.

6) Going forward, would the authors propose the same workflow? Would not be better to test for exposure (e.g. serology, PCR) and then conduct xenodiagnosis on seropositive or PCR positive animals?

It would be great to first test for infection and/ or exposure and then conduct xenodiagnosis on seropositive or PCR positive animals. Due to the logistics of getting vehicles and manpower for fieldwork and transport back to the lab, this was not feasible (to test serology and qPCR, blood samples need to transport to our main laboratory which is about 400 Km from endemic district and after diagnosis it is difficult to trace back those positive animals after a week as proper tagging is not available) in the future we will continue this same work flow.

7) Was it possible to get skin samples from the animals and test these for PCR? Would this be a better sample if one aims to assess their capacity as reservoirs?

We are thankful to the reviewer for the observation as leishmaniasis is dermotropic in nature and it is really important to look skin parasite burden in transmission studies. The animals include in this study (livestock) that have a unique religious and economic stature within India. In addition to the IACUC protocol, we assure the safety and comfort of these animals and approval from head of households during these studies. It is not possible to get skin samples from these animals, especially livestock. We had splenic biopsies of rodents to check for parasite burden.

**Reviewer #2**:

1. Lines 101-103: The authors should also refer to the *L. donovani* foci in Africa, as closely related to the ISC *L. donovani*, and the suspected reservoir hosts.

The authors agree with the reviewer and as suggested we have added comments to reflect this in the revised manuscript.

2.      Section 1.1.: it would be interesting if the authors indicated the vector control measures in the studied areas, if any, and if known.

We have already added the comments regarding ongoing vector control programs in endemic village by government in manuscript.

3.      Line 144: what is an m-tube vial? Explain or use manufacturer name.
4.      Line 151: This should be in section 1.5. In any case, the “poor condition” should be explained.
5.      Line 152: Indicate how the rats were euthanized.
6.      Lines 160-161: Explain why this number of sand fly females per animal. Is this enough to detect infection? What are the references for this?

The authors agree with the reviewer and as suggested we have added comments to reflect this in the revised manuscript with references.

7.      Line 162: Was the xenodiagnosis repeated throughout the year on rodents as well, or just livestock? If also on rodents, provide more detail.

We have revised the manuscript as suggested by reviewer.

8.      Lines 163-164: Explain to the non-expert reader why female sandflies were kept for 48 hours post-feeding.
We have added the suggested information in the revised manuscript.

9.      Line 166: Indicate at what temperature the samples were kept.
10.     Section 1.7.: The reference for the qPCR (ref. 44?) should be given from the first sentence and, certainly, for the primers used.

We have added the suggested information and references in the revised manuscript

11.     Section 1.8.: A reference must be given to the rk39 ELISA, and it should be stated the origin or preparation of the rk39 antigen used. Furthermore, at the end of the section, it is stated that rK39 rapid tests were conducted, so the title of the section should be changed accordingly, and the authors should clarify if the ELISA and the rapid tests were done to all samples, and if not, to which samples and the selection algorithm.

We have added the suggested information and changes were made in the revised manuscript accordingly.

12.     Lines 220-226: This belongs in the Introduction or the Discussion.

We have revised the manuscript as suggested by reviewer.

13.     Lines 225-226: Techniques, rather than technique. Indicate clearly if they are recommended together, or if only one is enough (“qPCR or rk39 ELISA”). In addition, qPCR is only meaningful together with the amplified target (or primers), so it should be indicated which qPCR is specifically recommended (with reference).

We have added needed information in revised manuscript

14.     Lines 226 and 228: “domestic animals” is not the best description for the sampled animals. It should be better “livestock and rodents”.

we have added the same in the revised manuscript

15.     Line 228: Exposure is how the results are interpreted. Here, the authors should simply indicate the percentage of animals with a positive serology result.
16.     Lines 228-233: There is no need to repeat all the results presented on Table 1. Plus, the values are in Table 1 and not Figure 3, as indicated, which shows the distribution of OD values.
17.     Line 239: Here should be the reference to Figure 3.
18.     Section 2.1.: It should be mentioned in the text the low positivity results for qPCR.
19.     Figure 3, Legend: it should be indicated that the test was an ELISA and what NE and E means.
20.     Lines 246-248: should be summarized to indicate that the authors have conducted xenodiagnosis to “quantify host-to-vector transmissibility and determining infectiousness of hosts” in endemic areas…
21.     Line 260: species name in italics.
22.     Lines 263-265: Both results should have been mentioned earlier in the Results.
23.     Ensure consistency, throughout, regarding the use of RDT (Methods) and ICT dipstick test (Discussion). The same name or abbreviation should be used for the same test as from the first mention.
24.     Also ensure consistency regarding the nomenclature for the animals: domestic animals, domestic cattle, livestock.

We thank the reviewer for comprehensive review. We have added the all the suggested information for above comments by reviewer in the revised manuscript.

25.     The Discussion should mention dogs, and their possible role or not in transmission in the ISC, as a complement to the animals surveyed in this work.

26.     The Discussion should compare these results with those obtained with other xenodiagnostic surveys of known *Leishmania* hosts, to evaluate how likely it would be to detect infected sandflies for each animal species surveyed, or demonstrate that the sample size for each animal species was adequate to detect infection.

The rationale for selecting specific numbers of different species of animals was provided in revised manuscript.

27.     The Discussion should be more explicit about the limitations of the work.

Changes were made in revised manuscript as suggested by reviewer.

**Reviewer #3**:

The manuscript entitled “Domestic Mammals as potential Reservoir Hosts for Leishmania donovani in India” describes an interesting study. The results of this study are of major eco-epidemiological importance and should lead to a better control of visceral leishmaniasis in the Indian Subcontinent. The manuscript should be published after taking into consideration the following comments:

Title: This study concerned not only domestic animals but also rodents living in the peri-domestic areas. In addition, the results provided strong evidence that investigated mammals are not reservoir hosts for *Leishmania donovani*. Thus, the title is misleading and therefore it should be change to “Mammals of the peridomestic areas in an endemic focus of VL are not reservoir host for *Leishmania donovani*”

We are thankful to the reviewer for this suggestion and have replaced the title as suggested also considering point 14 from reviewer 2. “**Livestock and rodents within an endemic focus of Visceral Leishmaniasis are not reservoir hosts for *Leishmania donovani”***

**Introduction**

Lines 72-73: The authors should differentiate between zoonotic visceral leishmaniasis due to *L*. *infantum* and anthroponotic visceral leishmaniasis due to *Leishmania donovani*.

Lines 102: In addition to canids, lagomorphs and wild rodents can also act as significant reservoirs.

The authors agree with the reviewer and as suggested we have added comments to reflect this in the revised manuscript.

**Methods**

I have a serious concern with the method.

Why the authors exclude dogs from the study? This is an important missing data.

48 hrs after blood feeding, engorged sandflies were stored in 70% alcohol for further DNA extraction and qPCR for *L. donovani*. The normal cycle of *L. donovani* within the sand fly takes 8-10 days to reach stationary phase. Why the authors did not allow for a full cycle accomplishment of *L. donovani* within the sand fly.

We sincerely appreciate the reviewer's points on excluding dogs from study and sand fly qPCR followed by 48hrs of incubation after xenodiagnosis. The authors have explained the reasons to exclude dogs from this study to R1. We have added comments to reflect this in the revised manuscript

We agree with the reviewer that normal cycle of *L. donovani* within the sand fly takes 8-10 days to reach stationary phase. Studies on parasite development in sand fly gut shows that after 48hrs of infected blood meal the peritrophic membrane had broken down and many procyclic promastigotes and nectomonads were readily visible. While by 72hrs, most of the bloodmeal had been digested and the bulk of the parasites had passed out with it. If there were low dose transmissions or poorly developed infections are established, it could be excreted out in early phase of development during blood meal digestion. We have standardized our xenodiagnosis protocol through infection experiments in our earlier articles to maximize our detection time limit. We have revised our manuscript with references.

**Results**

Since xenodiagnosis was repeated throughout the year, the authors should report results at each time.

We sincerely appreciate the reviewer's point on our findings. We have postulated the statement based on seasonal dynamics of *Leishmania* infection/ transmission as sand fly activity are reported mainly in March-August. We have modified our sentence accordingly in revised manuscript.

**Discussion**

The authors should report studies concerning xenodiagnoses performed on humans.

Taking into account that dogs are considered as reservoir hosts for *L. donovani* (Jumbulingam et al., 2017), the authors should discuss the rationale of not testing dogs.

We are thankful to the reviewer for the valuable suggestion and have now addressed about xenodiagnosis studies conducted on humans as suggested by the reviewer in the revised manuscript.
