# Supplementary material for: Livestock and rodents within an endemic focus of Visceral Leishmaniasis are not reservoir hosts for Leishmania donovani
Source: PLoS Negl Trop Dis. 2022 Oct 20;16(10):e0010347. doi: 10.1371/journal.pntd.0010347 (PMC9624431; doi:10.1371/journal.pntd.0010347)
Supplement: Attachment — Submitted filename: Revision 2 Response.docx [file pntd.0010347.s003.docx]

Reviewer #2: The authors have addressed many issues by the reviewers, but not all were completely integrated in the revised manuscript, or I was not able to identify where. Ensure that in the new response to reviewers includes the new or revised text and where the previous and new issues were addressed in the manuscript.

**Major issues**
1.      Despite the large number of sandflies used for xenodiagnosis, only 3 mammals had detectable *Leishmania* DNA, and it is not clear in the manuscript if these animals were included in the xenodiagnosis. Even if all seropositive mammals were infected, it is not given the percentage of the 192 animals that were seropositive, and it would be only 14% of all animals.

**Response:** We are thankful to the reviewer for the valuable suggestion and have now addressed the all 3 mammals have detectable parasitemia and 17 seropositive animals out of 192 included for xenodiagnosis. we have added the required information in revised manuscript (Page 12, Line 262-264)**.** All of the 192 reported animals had xenodiagnosis performed.
2.      The authors do not mention the estimated sensitivity of xenodiagnosis, and they should, but it would make more sense to concentrate the xenodiagnosis effort on animals with more likely *L. donovani* infection (high rk39 titre or positive qPCR), in terms of number of sandflies, but to conduct xenodiagnosis at different times during the day or along a week, for example. It is understandable if the authors have done the xenodiagnosis without prior knowledge of the infection status of the animals, but this should be discussed as a limitation of this work.

**Response:** Thank you for understanding that in these village settings, repeated sampling to check diagnostic status, and then return to the village to perform xenodiagnosis was not possible. We performed a sample size calculation with the assumption that less than 2% of animals would be qPCR positive to be able to find some parasitemic mammals. Our calculation indicated that we needed to sample 200 animals, and we sampled 255, but xenodiagnosis was performed on 192, finding roughly 1% of animals parasitemic. The consideration is not so much “the sensitivity” of xenodiagnosis, but how frequently are these exposed, infected animals likely to be xenodiagnosis positive. The act of being xenodiagnosis positive is a result of host immunity and resulting parasite clearance, sand fly host preference and dermatotrophism of the parasite in these animals, all of which contribute to the parasite available for detection in yet incompletely understood ways. This data is the first attempt to assess this, so using a basis of xenodiagnosis sensitivity for these species to perform the studies was not previously possible.

3.      The authors provide quite a lot of the information asked by reviewers in reply to reviewers, but not enough in the manuscript for many issues, including selection of mammals for xenodiagnosis, or the use of skin samples, which should be included in the discussion. Review throughout questions and answers to reviewers.

**Response:** We are thankful to the reviewer for the observation as leishmaniasis is dermotropic in nature and it is really important to look skin parasite burden in transmission studies. The animals include in this study (livestock) that have a unique religious and economic stature within India. In addition to the IACUC protocol, we assure the safety and comfort of these animals and approval from head of households during these studies. It is not possible to get skin samples from these animals, especially livestock. We have addressed all the suggested information in revised manuscript (Page 14-15, Line 328-333).

4.      Given that the authors have first conducted xenodiagnosis and only after detection of infection, the Results should reflect this order, including data that shows an estimation of how many animals subjected to xenodiagnosis were likely infected.

**Response**: We thank the reviewer for comprehensive review. We have added the all the suggested information for above comments by reviewer in the revised manuscript (Page 11-12, Line 260-265)**.**

5.      In my opinion, the authors have not addressed the question by all three reviewers regarding dogs as possible reservoirs of *L. donovani* in the ISC (or not), considering their role elsewhere, including Sudan, where *L. donovani* is also present, and the reasons why dogs were not included here.

**Response**: We sincerely appreciate the reviewer's points on excluding dogs from study. Our initial objective was to assess the role of livestock and rodents in endemic areas in relation to transmission as livestock density can play an important role in *Leishmania* epidemiology. Livestock are an important part of households in endemic setting of ISC. We agree that dogs are key reservoir hosts for the zoonotic transmission cycle and other findings in ISC also concluded that dogs are infected with *Leishmania donovani* and could be reservoir host. We are actively working in this area including serology and molecular identification of *Leishmania* infection in dogs as well as clinical investigations and its relation to infectiousness. As there is much more known about how dogs might present clinicopathologically with *Leishmania* infection, although there is some parallel data to that found in this manuscript reporting predominantly *Leishmania* exposure in livestock, there is much additional data required for evaluation of canine leishmaniosis. We are in the process of preparing to share our findings with the *Leishmania* research community very soon.

**Minor issues**:
1.      Line 61 – indicate that *P. argentipes* is the vector of *L. donovani* in India, to explain why this species was used, either here or elsewhere in the Author Summary.

**Response**: We appreciate the reviewer suggestion. We explained about it in the author summary section and in introduction section too (Page 3, Line 61-62 & Page 5, Line 102-105).
2.      Line 66 – indicate which population. Domestic animals?

**Response**: we have mention in revised manuscript (Page 3, Line 66).
3.      Lines 74 to 75 – “…and notable prevalence of Leishmania in more than 98 countries and territories” – a verb seems to be missing and this part of the sentence doesn’t follow well from the initial part, which refers to L. donovani, but the 98 countries are for any species that cause leishmaniasis in humans. This should rather follow or add to the first sentence of the Introduction.

**Response**: We are thankful to the reviewers for the observation and we have made the necessary changes suggested by the reviewers in the revised manuscript (Page 4, Line 70-75)
4.      Line 86 – Given that leishmaniasis is a vector-borne disease, the mammal host isn’t considered infectious. It is better to use “reservoir” here. The expression “that promote transmission” is not accurate either, as vectors actively seek to feed on mammals (infected or not). Finally, “to others” is not clear enough. Simply state that in other regions both humans and dogs are reservoirs unlike in India, or that are sources of infection to vectors.

**Response**: We are thankful to the reviewer for this suggestion and have replaced the sentence as suggested (Page 4, Line 85-86).
5.      Line 92 – define what “normal” is intended to mean in this sentence, or replace with a more adequate word. Does it refer to number of cases in relation to those recorded in previous years? Or does it refer to the actual number of cases during the outbreak?

**Response**: We appreciate the reviewer suggestion. It refers to new number of cases in relation to those recorded in previous years. We have revised the sentence in the manuscript (Page 4-5, Line 94-97)
6.      Lines 147 to 148 – Show calculations or indicate calculator used in the manuscript.

**Response:** We have revised and added the suggested information in the manuscript (Page 7, Line 149-153)
7.      Line 155 – indicate model and manufacturer of traps. (Page 7, Line 157-158)
8.      Line 156 – define proximity in distance range. (Page 7, Line 158-159)
9.      Lines 157 to 161 – it is only mentioned that blood was taken from rats, and that they were euthanized. Indicate what happened to the shrews and the voles here, rather than in section 1.5. (lines 174-176), for clarity.

**Response:** Blood was taken from all the rodent species. We have revised the sentence accordingly as suggested by reviewer (Page 7, Line 161)
10.     Lines 166 to 169 – This sentence is somewhat confusing. I think the issue is the placement of commas to indicate separate sections. I suggest: “… to limit the number of female P. argentipes per meal to 30-35, plus 10 males, and expose two feeding cups (…). The females used were three to five-day old (mature) and 12-hour starved.”

**Response:** We have revised the sentence accordingly as suggested by reviewer (Page 8, Line 170-172).
11.     Lines 171 to 172, which indicate 30-35 flies, seem to contradict lines 166 to 169, which state 60-70 flies.

**Response**: We limit the 30-35 flies per feeding cup and we used 2 feeding cup per animal, that’s why the cumulative number is 60-70 flies per animal. We revised the sentence (Page 8, Line 169-171).
12.     Line 181 – “ethanol” rather than “alcohol”, unless another alcohol was used, but then specify which. We have made this change (Page 8, Line 184)
13.     Line 184 – indicate the weight or volume used of the biological material for DNA extraction. We indicate (Page 8, Line 190).

14.     Lines 186 to 187 – What is this precipitation time? As far as I’m aware there is no precipitation step using the QIAmp kits, unless to determine DNA length. If that was the case, explain that this was done, but also indicate what was the time used, not just “increased”.

**Response**: We have revised the sentence and added the suggested information (Page 8-9, Line 188-192).
15.     Section 1.6. Indicate if the elution volume and eluent was the same for both kits, and if the quality assessment was also done for both methods of DNA extraction. If not, ensure that the method description is equally complete for both methods.

**Response**: Elution volume for both kits were different. We have added required information in revised manuscript (Page 8-9, Line 186-194).
16.     Line 209 – add “L. donovani” before rk-39. (Page 9, Line 212)
17.     Section 1.8 – Volumes for all ELISA components should be given, not just of the sample serum. (Page 10, Line 212-225)
18.     Line 235 – indicate how many of each livestock and rodents. (Page 11, Line 238-239)
19.     Line 237 – indicate in which groups of mammals Leishmania DNA was found. (Page 11, Line 239-241)
20.     Line 237 – indicate if qPCR positive animals were also rk39 ELISA positive or not. (Page 11, Line 241-242)
21.     Line 239 – indicate %s for each group as well. (Page 11, Line 243-244)
22.     Lines 243 to 250 – indicate that the comparison is between rk39 ELISA average OD, presumably, or what was compared. Also provide the values under comparison.

**Response**: The comparison is between the rk39 average OD of non-endemic control sera. We have revised the information in the manuscript (Page 11, Line 251-255).
23.     Figure 3 – it would make more sense to have the groups of animals in the same order as Table 1.

**Response**: We have rearranged the figure 3 as per reviewers’ suggestion in the manuscript.

24.     Lines 250 to 252 – Please clarify is sentence. Is the comparison between seropositive and seronegative cattle? Or are “other animals” other groups? In any case, it’s not clear what is being compared. Is it average OD?

**Response**: We have comparing seropositive cattle average OD to other mammal group (goats and rodent) with lower seroreactivity to *L. donovani* rk-39 antigen. We revised the sentence in the manuscript (Page 11, Line 256-257).
25.     Lines 256 – how were the 192 animals selected? What were the criteria used? – it should be explained in Materials and Methods. Were the three mammals with detectable Leishmania DNA included in the xenodiagnosis?

**Response**: For selecting the animals, inclusion criteria were either sex, mature/adult animals. Livestock were selected for xenodiagnosis only with prior approval from the head of household. We have explained this in sampling procedure section (Page 7, Line 151-152). Yes, all three mammals with detectable parasitemia were included in xenodiagnosis and this information is mentioned in revised manuscript (Page 11-12, Line 260-264).
